# Transmission of Similar Mcr-1 Carrying Plasmids among Different *Escherichia coli* Lineages Isolated from Livestock and the Farmer

**DOI:** 10.3390/antibiotics10030313

**Published:** 2021-03-17

**Authors:** Joaquim Viñes, Anna Cuscó, Sebastian Napp, Julio Alvarez, Jose Luis Saez-Llorente, Montserrat Rosàs-Rodoreda, Olga Francino, Lourdes Migura-Garcia

**Affiliations:** 1 Servei Veterinari de Genètica Molecular (SVGM), Universitat Autònoma de Barcelona, 08193 Barcelona, Spain; joaquim.vines@vetgenomics.com (J.V.); olga.francino@uab.cat (O.F.); 2 Vetgenomics, Edifici EUREKA, Parc de Recerca de la UAB, Campus UAB, 08193 Barcelona, Spain; anna.cusco@vetgenomics.com; 3 IRTA, Centre de Recerca en Sanitat Animal (CReSA, IRTA-UAB), Campus UAB, Universitat Autònoma de Barcelona, 08193 Barcelona, Spain; sebastian.napp@irta.cat; 4 OIE Collaborating Centre for the Research and Control of Emerging and Re-emerging Swine Diseases in Europe (IRTA-CReSA), 08193 Barcelona, Spain; 5 Centro de Vigilancia Sanitaria Veterinaria (VISAVET), Universidad Complutense, 28040 Madrid, Spain; jalvarez@ucm.es; 6 Departamento de Sanidad Animal, Facultad de Veterinaria, Universidad Complutense, 28040 Madrid, Spain; 7 Area de Programas Sanitarios y Zoonosis, S.G. de Sanidad e Higiene Animal y Trazabilidad, Ministerio de Agricultura, Pesca y Alimentación, 28014 Madrid, Spain; jsaezllo@mapama.es; 8 Departament d’Agricultura, Ramaderia, Pesca i Alimentació, Servei d’Alimentació Animal i Seguretat de la Producció Ramadera, 08007 Barcelona, Spain; montse.rosas@gencat.cat

**Keywords:** *Escherichia coli*, colistin, mcr, plasmids, MinION nanopore, hybrid sequencing, livestock

## Abstract

Colistin use has mostly been stopped in human medicine, due to its toxicity. However, nowadays, it still is used as a last-resort antibiotic to treat hospital infections caused by multi-drug resistant Enterobacteriaceae. On the contrary, colistin has been used in veterinary medicine until recently. In this study, 210 fecal samples from pigs (*n* = 57), calves (*n* = 152), and the farmer (*n* = 1) were collected from a farm where *E. coli* harboring *mcr*-1–*mcr*-3 was previously detected. Samples were plated, and *mcr*-genes presence was confirmed by multiplex-PCR. Hybrid sequencing which determined the presence and location of *mcr-1*, other antibiotic resistance genes, and virulence factors. Eighteen colistin resistant isolates (13 from calves, four from pigs, and one from the farmer) contained *mcr*-1 associated with plasmids (IncX4, IncI2, and IncHI2), except for two that yielded *mcr*-1 in the chromosome. Similar plasmids were distributed in different *E. coli* lineages. Transmission of *mcr*-1 to the farmer most likely occurred by horizontal gene transfer from *E. coli* of calf origin, since plasmids were highly similar (99% coverage, 99.97% identity). Moreover, 33 virulence factors, including *stx2* for Shiga toxin *E. coli* (STEC) were detected, highlighting the role of livestock as a reservoir of pathotypes with zoonotic potential.

## 1. Introduction

The vast majority of antimicrobials used in veterinary medicine are also used in human medicine. The consumption of antimicrobial agents has increased the selection of resistant bacteria in both human and veterinary medicine. Additionally, the presence of resistance genes in mobile genetic elements has probably played a major role in the inter- and intra-species transmission of antimicrobial resistance. 

Use of colistin in human medicine has been abandoned, due to its toxicity when applied systemically. Nevertheless, nowadays, the emergence of multidrug-resistant (MDR) Gram-negative bacteria in hospital settings has left no other choice but to use colistin as the last-line treatment option despite its toxicity. Contrarily, in veterinary medicine, colistin sulfate has been used orally for many decades to treat infections caused by *Enterobacterales* [1]. In particular, colistin tablets are available for calves in many countries for the prevention and treatment of neonatal colibacillosis [2]. Additionally, studies performed in different EU countries have reported the prophylactic and metaphylactic use of colistin for the prevention and treatment of enteric diarrheas in pigs [3,4,5,6]. In this scenario, Spain was the country with the highest sales of colistin for food-producing animals in the EU in 2014 [7]. Fortunately, colistin consumption has been drastically reduced in the last years after the implementation of a specific program “Reduce Colistin”, targeting pig production with the voluntary agreement of producers [8]. 

Until 2015, resistance to colistin was only associated with chromosomal mutations. More recently, different plasmid-mediated mechanisms conferring resistance to colistin have been identified [9,10,11,12,13], with the most prevalent one, *mcr*-1, being distributed worldwide [14]. The emergence of colistin resistance in mobile genetic elements has raised the concern of the scientific community, since the transmission of resistance from farm to fork could further complicate the treatment of severe infections in human hospitals.

Back in 2017, the co-occurrence of *mcr*-1 and *mcr*-3 was described for the first time in Spain in an *Escherichia coli* of calf origin [15]. This isolate was obtained from a fecal sample at a slaughterhouse in the frame of the Spanish National Monitoring Program for antimicrobial resistance carried out in 2015. The farm of origin of the calf was identified, and a visit was carried out in September 2017 to sample the premises and to determine if this *E. coli* genotype was endemic in the farm. In this context, whole genome sequencing combining Nanopore and Illumina technologies was applied to study the dynamics of the transmission of colistin resistance within the livestock and the farmer, the characterization of plasmids, the location and genomic context of resistance genes, and the detection of virulence factors.

## 2. Results and Discussion

Visible growth on MacConkey agar supplemented with colistin was observed for 18/210 fecal samples. The multiplex PCR confirmed the presence of *mcr*-1 in the 18 *E. coli* isolates (13 from calves, four from pigs and one from the farmer). No additional *mcr*-gene variants were detected. 

### 2.1. Antimicrobial Susceptibility Testing

MIC values for colistin varied, with one isolate exhibiting a MIC of 2 mg/L, 15 isolates equal to 4 mg/L, and two showing a MIC ≥ 8 mg/L (Table S1). Furthermore, all the colistin-resistant isolates were also resistant to ampicillin, ciprofloxacin, streptomycin, chloramphenicol, sulfamethoxazole, and trimethoprim. Additionally, 17 isolates exhibited resistance to tetracycline, 16 to nalidixic acid and florfenicol, 14 to kanamycin, and 13 to gentamicin. Finally, phenotypic resistance to cefotaxime was observed in three isolates, whereas resistance to ceftazidime was detected in two (Table S1). All the isolates were MDR.

The 18 *mcr-1* positive isolates were sequenced. Chromosome size ranged from 4,613,927 bp (Farmer) to 5,586,543 (calve 15B_22), with an average size of 5,009,072 bp (Table S2). Completeness ranged from 91.9% (P2_16) to 99.8% (15A_11 and 14_4), with an average of 98.5%, except for the isolate P2_2 (76.1%). The genome size and CDS number were similar when compared with the values obtained for phylotype A reference (NC_000913.3) and for phylotype B1 reference (NC_018658.1).

**Figure 1:**
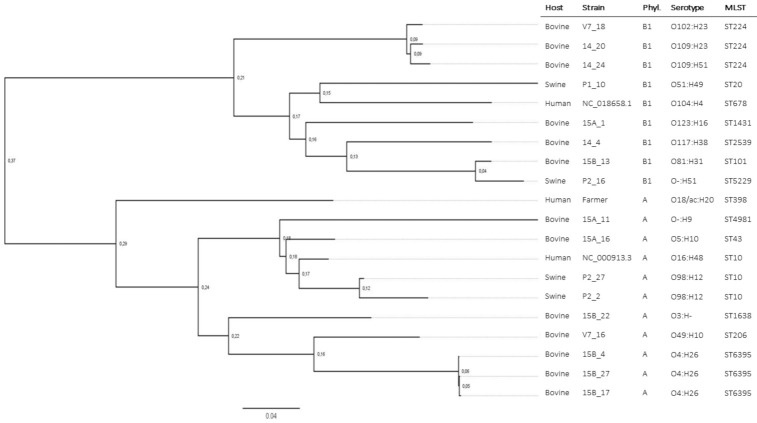
Chromosome phylogeny based on Single Nucleotide Polymorphisms (SNPs) retrieved with CSIPhylogeny and visualized with FigTree. Isolates are clustered according to phylotype and serotype. Two *E. coli* references were included that belonged to different phylotypes: NC_018658.1 for phylotype B1, and NC_000913.3 for phylotype.

Ten isolates belonged to phylotype A (seven from cattle, two from swine, and one from the farmer), and eight to phylotype B1 (six from cattle and two from swine). Phylogenetic analysisbased on Single Nucleotide Polymorphisms (SNPs) clustered isolates from phylotype A and B1 separately (Figure 1). The most represented multi–locus sequence type (MLST) was ST6395 (three isolates from calves) and ST224 (three isolates from calves), followed by ST10 (two swine isolates). The ST6395 isolates shared the same serotype (O4:H26), as well as the ST10 isolates (O96:H1) (Table S2).

**Figure 2:**
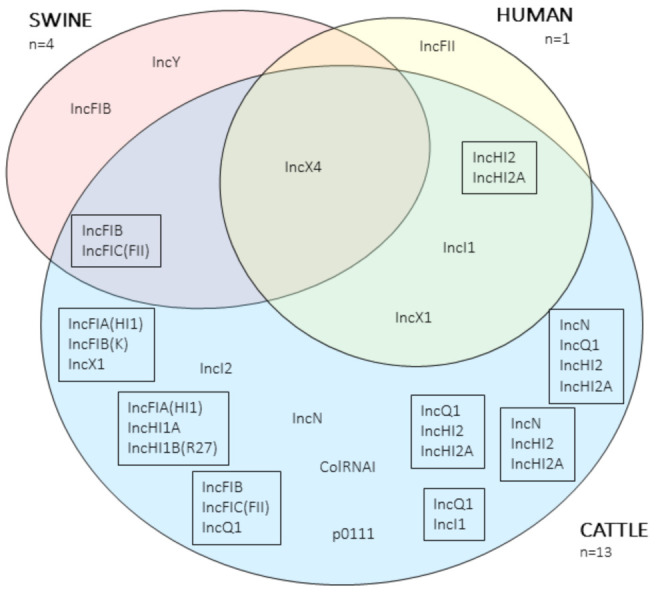
Venn diagram representing the replicons of the antimicrobial resistance gene (ARGs) plasmids. Replicons inside a box indicate a combination of replicons within the same plasmid.

A total of 48 plasmids bearing antimicrobial resistance genes (ARGs) (including those encoding for *mcr*–1) were retrieved after sequencing and assembling: 36 plasmids from the bovine isolates (ranging from one to five per isolate), seven from porcine (from one to two per isolate), and five from the farmer’s isolate (Table S3). Several replicons were identified in these plasmids, some of them in the same mobile genetic element, and 19 different replicon combinations (Figure 2). The most prevalent replicon was IncX4 (*n* = 14), which harbored the *mcr-1* gene and was present in isolates from the three hosts considered. Other common replicons were IncFIB (*n* = 9), IncHI2 / IncHI2A combination (*n* = 7), IncFIC (*n* = 6), and replicon combination IncFIB / IncFIC (*n* = 6).

### 2.2. Antibiotic Resistance Genes

**Figure 3:**
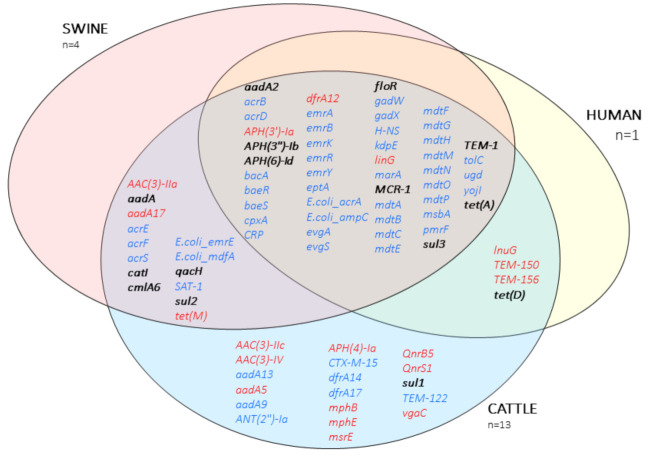
Venn diagram of the antibiotic resistance genes described in this study. Text in blue, chromosomal location; text in red, plasmid location; bold, located either in the chromosome or plasmid.

A total number of 85 ARGs were identified using Abricate with CARD (Table S3), encoding resistance to different antibiotic classes; cephalosporins, beta-lactams, aminoglycosides, fluoroquinolones, lincosamides, macrolides, peptides, phenicols, sulfonamides, tetracyclines, and trimethoprims among others. All the isolates were classified as MDR genotypically. Furthermore, all 85 ARG were encountered in isolates of cattle origin, while 63 of them were found in pigs and 53 in the human isolate (Figure 3).

Regarding the localization of the ARGs, 51 were located exclusively in the chromosome, 19 in plasmids, and 15 either in the chromosome or in plasmids (Figure 3).

After the in-silico analysis, *mcr*–1 gene was not found in one isolate of calf origin (15A_11), even though it had tested positive by PCR. This isolate contained the *bla*_CTX-M-15_ gene located in the chromosome. Upstream CTX-M-15 was a complete IS3 element and Δ*tnpA* from the ISEcp1 element, while downstream, there was Δ*tnpA* from the Tn2 element.

### 2.3. Genetic Context of mcr-1

Of the 17 isolates in which the *mcr-1* gene was identified by WGS, 15 carried the *mcr*-genes in plasmids (14 IncX4, one IncHI2, and one IncI2). Isolate 15B_22 contained two copies of the gene in two different plasmids, IncX4 and IncHI2 / IncHI2A. The remaining two isolates harboured the gene inserted in the chromosome (15B_13 and P2_16).

**Figure 4:**
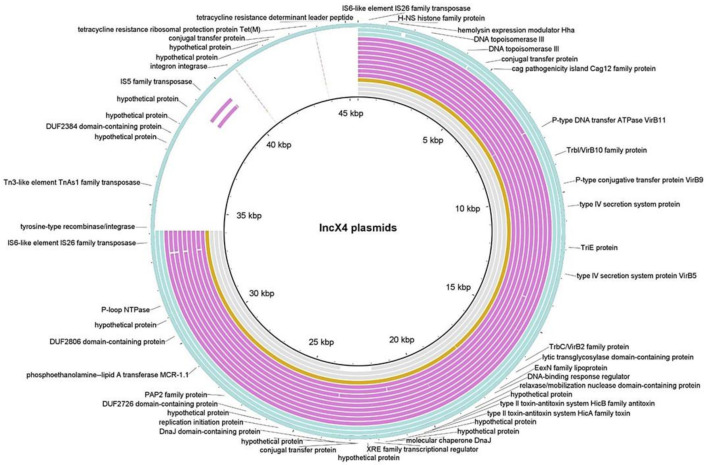
BLAST Ring Image Generator (BRIG) visualization of the 14 IncX4 plasmids from this study and three IncX4 plasmids from NCBI: pEC11b, pMCR1-NJ-IncX4, and pP744T-MCR1. Rings from outside to inside: P1_10, P2_2, P2_27 (blue); V7_18, V7_16, 15B_4, 15B_27, 15B_22, 15B_17, 15A_16, 14_4, 14_24, 14_20 (pink); farmer (orange); pP744T-MCR1, pMCR1-NJ-IncX4, pEC11b (grey). IncX4 plasmid from P1_10 is shown as the reference (longest sequence), with an extra-region of approximately 12,000 bp that is flanked by two IS26 elements and harbors the *tetM* gene conferring resistance to tetracycline. Isolates 15B_27 and 15_17 presented IS5 transposase, as P1_10 (pink fragments near 40 kb location for P1_10).

Four different environments for the *mcr-1* gene were found within these isolates (Table 1). All of these constructs have been previously described in other studies [16]. The main IncX4-plasmid backbone was present in all IncX4 plasmids (Figure 4 and Figure S1 and Figure S2). The IncX4 plasmid from the farmer shared the highest identity and coverage with their counterparts from calves (99.97% and 99%, respectively, Table 2). While 13 of the 14 IncX4 plasmids were approximately 33–35 kbp and had a GC content around 42%, the lncX4 plasmid from isolate P1_10 was larger (45,441 bp), and with higher GC content (44.1%). The latest harbored the *tetM* gene conferring resistance to tetracycline. Two IS26 elements were flanking this extra-region of approximately 12,000 bp. All IncX4 plasmids carried the type IV secretion system (T4SS), allowing the plasmid to be self-transmissible, and the HicAB toxin-antitoxin system for plasmid maintenance and stability. 

IncI2 and IncHI2 plasmids presented a size of 61,766 bp and 234,156 bp, respectively (File S1 and File S2, respectively). Both plasmids contained the conjugative mechanism T4SS and the replication machinery. While IncHI2 yielded HipA toxin-antitoxin system, IncI2 plasmid harbored RelE/ParE, Hok, and HicAB toxin-antitoxin systems. Finally, the IncHI2 plasmid contained *mcr*-1 together with six other AMR genes; *aadA2*, *aph(3′’)-Ib*, *aph(6)-Id*, *dfrA12*, *floR*, and *tetM*. 

### 2.4. Virulence Factors

**Figure 5:**
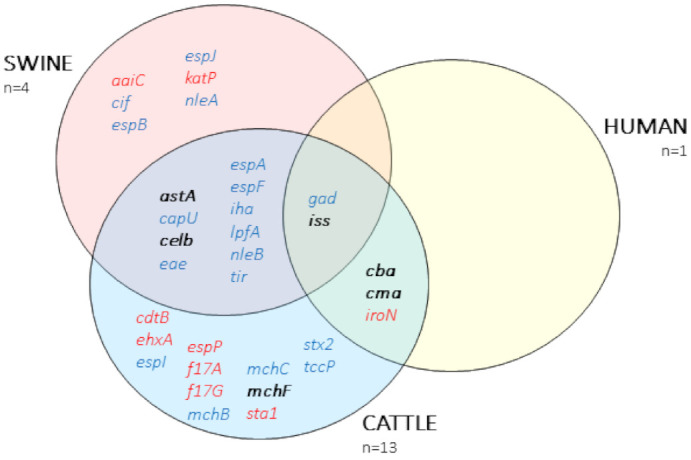
Venn diagram representing the virulence factors described by VirulenceFinder. Text in blue, chromosomal location; text in red, plasmid location; bold, located either in the chromosome or plasmid.

A total of 33 virulence factors were detected (Table S3). While nine genes were found exclusively in plasmids, 18 were located in the chromosome. The remaining six virulence genes were found in both locations, chromosome and plasmids. In general, *E. coli* from cattle origin contained the highest amount of virulence genes (27) (Figure 5), followed by swine (18) and the isolate from the farmer (5). 

A total of 13 plasmids of mainly two replicon families, IncF (*n* = 11) and Col (*n* = 2) harbored these virulence genes (IncF-plasmids information in Table S4). 

Some of these virulence factors conferred different pathotypes, such as adherence factors *eae* (intimin), *tir* (translocated intimin receptor), *f17A* (major F17 fimbriae subunit), *f17G* (adherence F17 fimbriae subunit); enzymes *katP* (plasmid-encoded catalase peroxidase), *ehxA* (enterohemolysin), *espP* (plasmid-encoded extracellular serine protease); secretion-related genes *espA* (type III secreted effector, needle sheath), *espB* (type III secreted effector, translocation pore), *nleA* (non-LEE encoded effector A), *nleB* (non-LEE encoded effector B); toxins *astA* (enteroaggregative heat-stable toxin, EAST-1), *sta1* (heat-stable enterotoxin ST-Ia), *cdtB* (cytolethal distending toxin subunit B), and *stx2* (Shiga toxin). 

Isolate 15B_13 of calf origin (phylotype B1, serotype O81:H31, ST101) harbored the *mcr-1* gene in the chromosome and contained *stx2* (*stx2A* and *stx2B* subunits) encoding for Shiga toxin. A complete phage D108 was found spanning a region of 86.6 Kbp that harbored the *stx2* gene. Additionally, this isolate also contained several virulence factors; *iha*, *lpfA*, *gad*, *iss*, *astA*, *cba*, *celb*, *mchB*, *mchC*, and *mchF*.

## 3. Discussion

The aim of this study was to conduct a cross-sectional thorough sampling of a farm where co-occurrence of *mcr*-1-*mcr*-3 *E. coli* was previously detected, and evaluate the transmission of colistin resistance plasmids within the farm applying WGS. Our approach using long-reads to assemble, and short reads to polish (hybrid assembly), allowed to close chromosomes and circular plasmids harboring *mcr-1* genes, and to study their location and genomic context among different *E. coli* lineages.

*mcr*-1 positive *E. coli* were detected in all host species present in the facilities, including one human (the farmer). According to the farm book, pigs and calves sampled in 2017 were orally treated with colistin. Going back through the farm book, batches of both pigs and calves reared between 2015 and 2017 were consistently prescribed colistin in the same phases of their production cycle. This management practice in terms of medication regime suggests a routine use of colistin in consecutive batches, facilitating the persistence of colistin resistance mechanisms. Additionally, phenotypic and genotypic resistance to other families of antimicrobials widely used in the farm were also detected, such as β-lactams, tetracycline, aminoglycosides, and sulfonamides. 

In agreement with previous studies [14,17,18,19,20,21,22], the 15 isolates bearing *mcr*-1 in mobile genetic elements were associated with IncX4, IncI2, and IncHI2 plasmids, with IncX4 being the most prevalent. All IncX4 plasmids shared the same backbone, and differences were due to inserted sequences. In the case of isolate P1_10, this insertion expanded 12 kbp and comprised the *tetM* gene. Additionally, isolate 15B_22, presented two plasmids encoding for *mcr*-1 [18,19,20] with IncHI2 plasmid carrying also resistance genes for tetracycline (t*etM)*, trimethoprim (*dfrA12*), aminoglycosides (*aph(3″)-Ib*, *aph(6)-Id*) and florfenicol (*floR*), as well as colistin (*mcr*-1). Different families of ARGs located in the same plasmid facilitated the persistence and selection of resistance to antibiotics not used in the farm. Even if colistin was withdrawn (as it happened after our visit to the farm), the use of doxycycline could co-select indirectly for the *mcr*-1 gene. 

As previously described [17,19,21,22,23], herein, *mcr-1* was also integrated into the genome in two isolates of different animal origins. In one of them, the *ISApl1* element was flanking *mcr-1* upstream and downstream, a structure that probably facilitated the movement of the whole element by transposition. The other isolate had lost the *ISApl1* element, establishing the *mcr-1* as a heritable trait overcoming any possible fitness cost of plasmid maintenance [24]. Furthermore, this isolate had become permanently resistant even when the selective pressure was removed [17,25].

Although phylogenetic analysis clustered the *E. coli* isolates in phylotype A and B1, there were different MLST linages harboring the colistin resistance genes in highly similar plasmids (identity ranging from 99.96% to 100%). The farmer’s isolate (ST398) did not match the MLST type of any of the livestock isolates. Interestingly, ST398 was the only ST shared between livestock and bloodstream infections in a study carried out across the United Kingdom, underlying the zoonotic potential of some *E. coli* linages [26]. Conversely, the lncX4 plasmid from the farmer’s isolate was highly similar to those obtained from calves (14-4, 14-20, 15A-16, 15B-22, and V7_18), sharing length, GC content, and genomic context (*mcr-1-pap2*). These results suggest the transmission of resistance through mobile genetic elements between different *E. coli* lineages from livestock to the farmer. Several studies have demonstrated the spread of ARGs from food-producing animals to veterinarians and personnel in direct contact with animals [27,28,29], highlighting the importance of implementing hygiene measures to reduce this transmission. Even though our approach was focused on detecting the presence or absence of *mcr*-1-*mcr*-3 genes by picking a unique colony per sample, further studies including within-host diversity of *mcr-1 E. coli* isolates should be performed to introduce the variability of the *E. coli* population within a sample.

All the isolates from this study exhibited a MDR profile. In addition, ARGs, as well as virulence factors, were described both in the chromosome and plasmids. *E. coli* isolates of cattle origin showed the highest number and diversity of plasmids encoding for both ARGs and virulence genes, including *stx2*. Shiga toxin *E. coli* (STEC) serotype O81:H31 bearing *stx2* have also been described previously [30] and are considered important foodborne zoonotic pathogens [30,31,32,33]. To our knowledge, this is the first description of a *mcr*-1 positive STEC of cattle origin and highlights the importance of food producing animals as reservoirs of ARG and virulence determinants. 

On the contrary, isolate 15A_11 resulted negative for the *mcr-1* gene after in silico analysis, even though previous PCR tested positive for this gene. Presumably, the isolate lost the *mcr*-1 plasmid during sub-culturing steps, resulting in a false-negative result. Besides, 15A_11 isolate (phylotype A, ST4981) was resistant to cephalosporins (cefotaxime and ceftazidime) with *bla_CTX-M-15_* inserted in the chromosome. Interestingly, upstream of the gene, there was an IS3 element, and downstream there was a truncated Tn2, indicating a possible recombination event, as has been previously described [34,35]. The acquisition of *bla_CTX-M-15_* in livestock is concerning, since it is widely disseminated [36,37,38], especially in healthcare facilities [38,39,40,41], and can compromise the treatment of Gram-negative infections.

Regarding the analyses of the sequencing data, it is important to be aware of the limitation of databases to obtain the most accurate results. On the basis of an example from this study, cytolethal distending toxin (CDT) is composed of three subunits (encoded by three genes, *cdtA*, *cdtB*, and *cdtC*), which are all required for bonding to the cell’s surface and for entering the cell. However, VirulenceFinder only found the *cdtB* gene, while *cdtA* and *cdtC* were also present when aligning the sequence with the reference genome from NCBI. In depth analysis of the sequencing data should consider if a virulence element is composed of different genes to be fully operative.

## 4. Materials and Methods

### 4.1. Study Design

After the identification in 2017 of an *E. coli* isolate of bovine origin harboring both *mcr-1* and *mcr-3*, the farm from where the sample was obtained was contacted by the Spanish Official Veterinary Services to follow up on the finding. This farm belonged to a private farmer and contained two separate areas. The first area was a farrow-to-finish system for pig production. At not more than a 100-m distance, and without a physical barrier, there was a multi-origin bovine fattening farm, also the property of the farmer.

The number of samples to be collected in each of the housing facilities (three housing facilities for bovine and one for swine) was calculated to allow for the detection of a prevalence of *mcr-1-mcr-3 E. coli* of at least 5%, with a 95% confidence level. Sample size calculations were carried out using the WinEpi tool (http://www.winepi.net/uk/index.htm, accessed on 1 March 2021). Additionally, the farmer was interviewed by the Official Veterinary Services, and the farm´s treatment book was inspected to determine the antimicrobial treatments prescribed to the two animal species from 2015 to the time of sampling.

A total number of 210 fecal samples were collected: 152 from calves (*n* = 501), 57 from fattening pigs (*n* = 900), and one from the farmer (*n* = 3). Fecal samples were taken from individual animals and transported to the laboratory at 4° C on the day of sampling. The farmer sent a refrigerated fecal sample by courier within 24 h of the visit to the farm. Faces were homogenized and plated onto both MacConkey agar and MacConkey agar supplemented with colistin (2mg/L). For quality control of the colistin plates, a positive and negative control were also included. Following incubation, one presumptive colistin resistant *E. coli* isolate per positive sample was identified by PCR [42] and stored at –80 °C for further analyses. Detection of the five *mcr* genes (*mcr-1* to *mcr-5*) described at the time of sampling was performed by multiplex PCR, as described by Rebelo et al., [43].

### 4.2. Antimicrobial Susceptibility Testing

Minimal inhibitory concentration (MIC) was carried out for 14 antimicrobial agents (VetMIC GN-mo, Swedish National Veterinary Institute) in those isolates harboring *mcr*-genes. Antimicrobials tested were ampicillin (1 to 128 mg/L), cefotaxime (0.016 to 2 mg/L), ceftazidime (0.25 to 16 mg/L), nalidixic acid (1 to 128 mg/L), ciprofloxacin (0.008 to 1 mg/L), gentamicin (0.12 to 16 mg/L), streptomycin (2 to 256 mg/L), kanamycin (8 to 16 mg/L), chloramphenicol (2 to 64 mg/L), florfenicol (4 to 32 mg/L), trimethoprim (1 to 128 mg/L), sulfamethoxazole (8 to 1,024 mg/L), tetracycline (1 to 128 mg/L), and colistin (0.5 to 4 mg/L). Epidemiological cut-off values were those recommended by the European Committee on Antimicrobial Susceptibility Testing (EUCAST). MDR isolates were defined as resistance to at least three different antimicrobial families [44].

### 4.3. Whole Genome Sequencing and Data Analysis

DNA from *mcr*-positive isolates was extracted using QIAGEN DNeasy^®^ Ultraclean Microbial Kit under manufacturer’s conditions. Illumina (San Diego, CA, USA) libraries were prepared by enzymatic fragmentation and double indexing using an NGSgo kit (GenDx, Utrecht, Netherlands), according to the manufacturer’s instructions. The indexed libraries were pooled, denatured, and diluted to a final concentration of 4 nM. The pooled library was sequenced on the MiSeq system (Illumina) with a 300-cycle MiSeq reagent kit v2. Illumina paired-end reads were merged into one unique file per isolate using a custom python script (https://github.com/isovic/racon/issues/68, accessed on 1 March 2021).

In parallel, DNA was quantified using Qubit dsDNA BR assay (Invitrogen by ThermoFisher Scientific), and sequenced using MinION sequencer (Oxford Nanopore Technologies, ONT, Oxford, UK) in two runs of 9 samples each. Two sequencing libraries were prepared using 400 ng DNA per sample with the Rapid Barcoding Kit (SQK-RBK004, ONT, Oxford, UK) according to the manufacturer’s instructions. The samples were run using MinKNOWN software (version 18.07.18). Fast5 files generated were basecalled and demultiplexed using Albacore v2.3. Reads classified as pass (minimum Phred score of 7) were used for further steps. A second round of demultiplexing was performed with Porechop [45], which also assisted to trim barcodes, chimeric reads, and sequencing adapters.

Nanopore fastQ files were used to perform the assembly of genomes and plasmids from the 18 isolates with Flye [46] (v2.6), specifying flags “--nano-raw”, and “--plasmids” to retrieve smaller contigs, such as plasmids. Once the assembly was finished, raw ONT reads were mapped to the contigs using minimap2 (v2.17) [47]. A first round of polishing using Racon [48] (v1.4.10) with ONT reads was performed, followed by two rounds of polishing with Medaka (v0.11.4), using the “medaka_consensus” option. A final round of polishing was performed using Illumina reads. First, Illumina reads were mapped to the ONT-polished contigs using Minimap2 (v2.17) and then a round of Racon (v1.4.10) was performed. The files obtained from this polishing step were the final assemblies and were used for the further analyses.

In order to characterize the presence of plasmid replicons, antibiotic resistance genes (ARGs) and virulence factors, Abricate [49] (v0.8.13) along with PlasmidFinder [50], CARD [51] and VFDB [52] (respectively) were applied, with a minimum identity and coverage of 90%. Insertion sequences, phage presence, and presence of conjugative elements were analyzed using ISFinder [53], PHASTER [54], and OriTFinder [55], respectively. Gene annotation was performed with NCBI Prokaryotic Genome Annotation Pipeline (PGAP) [56] and Prokka [57] (v1.14.16). BUSCO [58] (v4.0.1) was used to assess genome completeness with the *Enterobacteriaceae* database (OrthoDb v10.1). SerotypeFinder [59], MLST [60], CSIPhylogeny [61] to call SNPs and ClermonTyping [62] were also applied. 

Contigs were visualized with Bandage [63]. Plasmid annotation was visualized with BLAST Ring Image Generator [64] (BRIG) and SnapGene Viewer (v5.0.7). Finally, phylogeny was visualized with FigTree (v1.4.3) (http://tree.bio.ed.ac.uk/software/figtree/, accessed on 1 March 2021).

## 5. Conclusions

In conclusion, co-existence of *mcr*-1-*mcr*-3 in *E. coli* from the farm could not be confirmed. Nevertheless, we isolated *mcr*-1 across the farm in different *E. coli* linages, mainly associated with plasmids of the IncX4, IncHI and IncHI2 families. The most likely mechanism for the farmer to have acquired the *mcr*-1 gene was the horizontal gene transfer from the calves, since plasmids from both origins were highly similar. Additionally, *mcr*-1 positive *E. coli* isolates were MDR and contained a high number of virulence genes, including *stx2*, demonstrating that food-producing animals can be a reservoir of such determinants, posing a risk for human health, especially for personnel at the farm.

## Figures and Tables

**Table 1:** Location and genomic context of *mcr-1* gene. *mcr-1* gene was found in 17 out of the 18 colistin resistant *E. coli* isolates either in a plasmid (14 in IncX4, one in IncI2, and one in IncHI2 replicons), or the chromosome. Isolate 15B_22 contained two plasmids with *mcr-1* gene (lncX4 and lncHI2). No correlation between phylotypes and specific genomic context were described. Phyl., phylotype; loc., location; Pl., plasmid; Chr., chromosome.

Host	Isolate	Phyl.	MLST	*mcr-1*	*mcr-1* loc.	Pl. GC%	Pl. Size (bp)	*mcr-1* Genomic Context
Human	Farmer	A	ST398	yes	IncX4	41.9	33,270	*mcr-1-pap2*
Swine	P2_16	B1	ST5229	Yes	Chr.	-	-	*mcr-1-pap2*
	P1_10	B1	ST20	yes	IncX4	44.1	45,441	*mcr-1-pap2*
	P2_2	A	ST10	yes	IncX4	42.5	35,296	*mcr-1-pap2*-ΔISApl1
	P2_27	A	ST10	yes	IncX4	42.5	35,326	*mcr-1-pap2*-ΔISApl1
Bovine	15B_27	A	ST6395	yes	IncX4	42.2	34,706	*mcr-1-pap2*-ΔISApl1
	15B_17	A	ST6395	yes	IncX4	42.2	34,758	*mcr-1-pap2*-ΔISApl1
	15B_4	A	ST6395	yes	IncX4	41.8	33,577	*mcr-1-pap2*-ΔISApl1
	14_24	B1	ST224	yes	IncX4	41.8	33,557	*mcr-1-pap2*-ΔISApl1
	V7_16	A	ST206	yes	IncX4	42.2	34,618	*mcr-1-pap2*-ΔISApl1
	15A_16	A	ST43	yes	IncX4	41.8	33,242	*mcr-1-pap2*
	14_20	B1	ST224	yes	IncX4	41.8	33,283	*mcr-1-pap2*
	14_4	B1	ST2539	yes	IncX4	41.8	33,262	*mcr-1-pap2*
	V7_18	B1	ST224	yes	IncX4	41.9	33,268	*mcr-1-pap2*
	15B_22 15B_22	A A	ST1638 ST1638	yes yes	IncX4 IncHI2	41.9 45.4	33,268 234,156	*mcr-1-pap2* ISApl1-*mcr-1-pap2*
	15A_1	A	ST1431	yes	IncI2	42.5	61,766	ISApl1-*mcr-1-pap2*
	15B_13	B1	ST101	Yes	Chr.	-	-	ISApl1-*mcr-1-pap2*-ISApl1
	15A_11	B1	ST4981	no	-	-	-	-

**Table 2:** Coverage and identity comparison of the farmer’s *mcr*-1-IncX4 plasmid versus livestock IncX4 plasmids.

Isolate ID	Coverage (%)	Identity (%)
P1_10	74	99.99
P2_2	94	99.87
P2_27	94	100
V7_16	95	99.97
15B_17	95	99.99
15B_27	95	100
15B_4	98	99.97
14_24	98	99.99
14_4	99	99.96
V7_18	99	99.96
15A_16	99	99.97
14_20	99	99.97
15B_22	99	99.97

## Data Availability

Nanopore and Illumina fast Q files, genomes and plasmids sequences, and supplementary material that support the findings of this study are openly available in OSFHome at http://doi.org/10.17605/OSF.IO/7Q2CB, reference number 7Q2CB.

## Supplementary Materials

The following are available online at https://www.mdpi.com/2079-6382/10/3/313/s1, Figure S1: Prokaryotic Genome Annotation Pipeline (PGAP) annotation of the IncX4 plasmid from the Farmer visualized with SnapGene Viewer; Figure S2: Prokaryotic Genome Annotation Pipeline (PGAP) annotation of the IncX4 plasmid from P2_2 visualized with SnapGene Viewer.Table S1: Minimal inhibitory concentration (μg/mL) for the 14 antibiotics tested. R, resistant; WT, wild type; Table S2: Chromosomal assembly and in-silico analyses of the colistin resistant E. coli isolates; Table S3: Antibiotic Resistance Genes (ARGs) and Virulence Factors (VFs) described by Abricate and CARD database; Table S4: IncF-family plasmids harboring virulence factors (VF) and antibiotic resistance genes (ARGs); File 1: GBK file with the annotation of the IncI2 plasmid harbouring mcr-1 gene from 15A_1.; File 2: GBK file with the annotation of the IncHI2 plasmid harbouring mcr-1 gene from 15B_22.
