# Supplementary material for: Transmission of Similar Mcr-1 Carrying Plasmids among Different Escherichia coli Lineages Isolated from Livestock and the Farmer
Source: Antibiotics (Basel). 2021 Mar 17;10(3):313. doi: 10.3390/antibiotics10030313 (PMC8003085; doi:10.3390/antibiotics10030313)
Supplement: Supplementary file 1 [file antibiotics-10-00313-s001.pdf]

SUPPLEMENTAL MATERIAL

Table S1

Minimal inhibitory concentration (µg/ml) for the 14 antibiotics tested. R, resistant; WT, wild type;

Table S2

Chromosomal assembly and in-silico analyses of the colistin resistant *E. coli* isolates. Genomes obtained by de novo assembled with nanopore long reads and polished with Illumina short reads. Phylotype, serotype and MLST were determined with ClermonTyping, and with SerotypeFinder and MLST from CGE-DTU. CDS were annotated with PROKKA and PGAP. BUSCO completeness with a total of 440 genes. Data from NCBI references for phylotyper Be and A correspond to NC_018658.1 and NC_000913.3 respectively. Abbreviations: C, BUSCO completeness (%);Ctgs, contigs;

Table S3

Antibiotic Resistance Genes (ARGs) and Virulence Factors (VFs) described by Abricate and CARD database. The legend is on the right side of the table. Chr., chromosome.

Table S4

IncF-family plasmids harboring virulence factors (VF) and antibiotic resistance genes (ARGs). Abbreviations: T4SS, type 4 secretion system.

Figure S1

Prokaryotic Genome Annotation Pipeline (PGAP) annotation of the IncX4 plasmid from the Farmer visualized with SnapGene Viewer.

Figure S2

Prokaryotic Genome Annotation Pipeline (PGAP) annotation of the IncX4 plasmid from P2_2 visualized with SnapGene Viewer.

File S1

GBK file with the annotation of the IncI2 plasmid harbouring *mcr*-1 gene from 15A_1.

File S2

GBK file with the annotation of the IncHI2 plasmid harbouring *mcr*-1 gene from 15B_22.
